# Prevalence and Severity of Spinal Osteoarthritis in Gout Patients Versus Non-Gout Controls

**DOI:** 10.3390/gucdd4020010

**Published:** 2026-04-30

**Authors:** Allyson Covello, Salim Zenkhri, Cheongeun Oh, Michael H. Pillinger, Michael Toprover, Fabio Becce

**Affiliations:** 1 Division of Rheumatology, Department of Medicine, New York University Grossman School of Medicine, 550 First Avenue, New York, NY 10016, USA; 2 Rheumatology Section, NY Harbor Health Care System, New York Campus, United States Department of Veterans Affairs, 423 E 23rd Street, New York, NY 10010, USA; 3 Department of Diagnostic and Interventional Radiology, Lausanne University Hospital, University of Lausanne, 46, rue du Bugnon, 1011 Lausanne, Switzerland; 4 Division of Biostatistics, Department of Population Health, New York University Grossman School of Medicine, 550, First Avenue, New York, NY 10016, USA

**Keywords:** gout, osteoarthritis, dual-energy computed tomography

## Abstract

Prior research suggests a connection between osteoarthritis and gout at sites commonly affected by gouty attacks. Whether this connection exists at sites with known monosodium urate crystal deposition but less commonly affected by gouty attacks, such as the lumbosacral spine, has not been previously investigated. We assessed whether lumbosacral osteoarthritis is more prevalent and more severe in subjects with gout compared with controls, and whether lumbosacral osteoarthritis is associated with higher levels of spinal monosodium urate deposition. Fifty gout subjects and 25 controls underwent dual-energy computed tomography imaging of the lumbosacral spine. We assessed lumbosacral osteoarthritis using a modification of a validated computed tomography scoring system, incorporating grade of intervertebral disc narrowing and facet joint osteoarthritis, and presence of spondylolysis and spondylolisthesis. We quantified spinal monosodium urate deposition using the default post-processing algorithm, plus a maximally specific algorithm to exclude potential artefacts. Forty-six gout subjects and 25 controls, average age 62 years, were included in the final analysis. Both gout and control subjects exhibited high rates of facet joint osteoarthritis and degenerative disc disease, with no difference in prevalence or severity between groups. Gout subjects did not have differing prevalence of spondylolysis and spondylolisthesis vs. controls. Subjects with lumbosacral osteoarthritis did not have higher levels of spinal monosodium urate deposition. Overall, lumbosacral osteoarthritis was not more prevalent or more severe in gout patients compared with controls, and spinal monosodium urate crystal deposition did not differ between patients with and without lumbosacral osteoarthritis.

## 1. Introduction

Osteoarthritis (OA), the most prevalent arthritis, affects 10–15% of adults in the United States and leads to significant quality of life impairment [1,2]. Gout, the leading cause of inflammatory arthritis, affects 5% of adults in the U.S. and similarly contributes to pain and functional limitation [3,4]. While OA was previously thought to be a condition of joint degeneration, additional research has shown that OA joints experience chronic, albeit low-level, inflammation [5]. Patients with gout experience more obvious acute inflammatory flares from monosodium urate (MSU) crystal deposition in joints, but they also have less recognized chronic inflammation, chronic joint disease, and systemic MSU deposition [6,7].

Clinicians have long observed an association between OA and gout. Imaging studies have demonstrated higher rates of knee OA in patients with gout [8,9], though this relationship has not been universal [10]. Additional research has shown a higher prevalence of foot and ankle joint changes consistent with OA, such as bone spurs, osteophytes, and cartilage degeneration, at sites of MSU crystal deposition, suggesting a possible pathophysiologic link between MSU deposition and OA [11,12]. However, no studies to date have investigated the relationship between gout, OA, and MSU deposition in joints commonly affected by OA but not by clinically apparent gout, such as the lumbosacral (LS) spine. Prior research by our group has shown MSU deposition in the spines of gout patients [6]. A higher prevalence and severity of LS OA in patients with gout, and a positive relationship between MSU deposition levels and LS OA, would further support a pathophysiologic link between OA and gout.

To investigate the relationship between gout and LS OA, we employed computed tomography (CT) to assess LS OA in gout and control subjects. To quantify LS OA, we used a modification of a previously defined imaging scoring system incorporating grade of intervertebral disc narrowing and facet joint OA, and presence or absence of spondylosis and spondylolisthesis. Secondarily, we employed dual-energy CT (DECT) to assess a possible relationship between spinal MSU deposition and LS OA. To measure MSU deposition, we used both a manufacturer’s default post-processing algorithm, and a maximally specific algorithm to exclude potential artefacts. We then performed cross-sectional and logistic regression analyses to assess whether gout patients had higher prevalence and severity of LS OA, and whether LS OA was associated with higher levels of MSU deposition. We hypothesized that patients with gout would have higher prevalence and severity of LS OA, and that patients with LS OA would have higher levels of spinal MSU deposition, indicating an association between gout and LS OA potentially mediated by MSU deposition.

## 2. Materials and Methods

### 2.1. Study Population

We analyzed previously collected data from a cross-sectional, age-restricted observational study that evaluated spinal MSU crystal deposition in gout patients versus controls using DECT. This original study found that MSU-coded volumes in the lumbosacral spines were significantly higher among the gout subjects vs. controls, even when using validated, maximally stringent post-processing settings to exclude potential artefacts [6,13]. The study enrolled 50 gout patients (25 with tophi, 25 without) and 25 non-gout controls recruited between September 2018 and March 2021 from rheumatology and primary care clinics at NYU Grossman School of Medicine, with referrals from the NY Harbor VA, Bellevue Hospital, and the Hospital for Special Surgery, and was approved by the NYU Grossman School of Medicine institutional review board. The original study was funded through an investigator-initiated research grant from Horizon Therapeutics (now Amgen).

Participants were aged 45–80 years. Gout subjects met ACR/EULAR classification criteria [14] and either had a serum urate (SU) ≥ 6.8 mg/dL off urate-lowering therapy (ULT) for ≥12 months, or SU > 6 mg/dL while on ULT for <6 months. Exclusion criteria for gout subjects included SU < 6.8 mg/dL without treatment, SU < 6 mg/dL with <6 months of ULT, or ULT use for >6 months regardless of SU. Individuals with other inflammatory arthritides or spinal malignancy were excluded. Controls were recruited from the same sites and met the same criteria, excluding gout-specific parameters.

### 2.2. Data Acquisition

Demographic data, medical history, surgical history, and Multidimensional Health Assessment Questionnaire [15] were collected. Low back pain was assessed using the Aberdeen low back pain scale [16]. All participants underwent physical examination including identification of peripheral tophi, serum chemistries, complete blood counts, erythrocyte sedimentation rate, C-reactive protein, and SU. Gout subjects were enrolled in a stratified fashion to ensure balanced representation of tophaceous and non-tophaceous disease.

All subjects underwent axial scanning of the LS spine from the thoraco-lumbar junction through the sacrum using a Somatom Force DECT system (Siemens Healthineers, Erlangen, Germany) [17]. Relevant data acquisition parameters were: tube potentials, 100 and 150 with tin filter (Sn150) kV; tube currents at 100 and Sn150 kV, 174–224 and 348–446 mA, respectively; pitch, 0.4; volume CT dose index, ~30 mGy. Image series were reconstructed at a section thickness/interval of 0.75/0.5 mm with a smooth kernel (Qr32d), resulting in an in-plane pixel size of ~0.3 × 0.3 to ~0.4 × 0.4 mm^2^.

### 2.3. Grading of Lumbosacral Osteoarthritis

To quantify subjects’ LS OA, we utilized a modification of a previously defined imaging scoring system incorporating grade of intervertebral disc narrowing and facet joint OA, and presence or absence of spondylosis and spondylolisthesis [18]. All assessments were made at the L1–L2 through L5–S1 levels. Two readers, a rheumatologist (4 years of experience) and a radiologist (6 years of experience) independently graded all subjects. Their grades were then adjudicated by a third reader, a musculoskeletal radiologist (19 years of experience). All readers were blinded to the clinical diagnoses of the subjects.

Degenerative disc disease was assessed in the sagittal plane on a scale of 0 to 3, where 0 = normal, disc height greater than the cephalad disc (except for the L5-S1 disc); 1 = disc height equal to the cephalad disc if it is normal, with no to slight disc bulging; 2 = disc narrower than the cephalad disc if it is normal (generally associated with disc bulging anteriorly and/or posteriorly); and 3 = severe narrowing, with end plates almost in or in contact.

**Figure 1.**
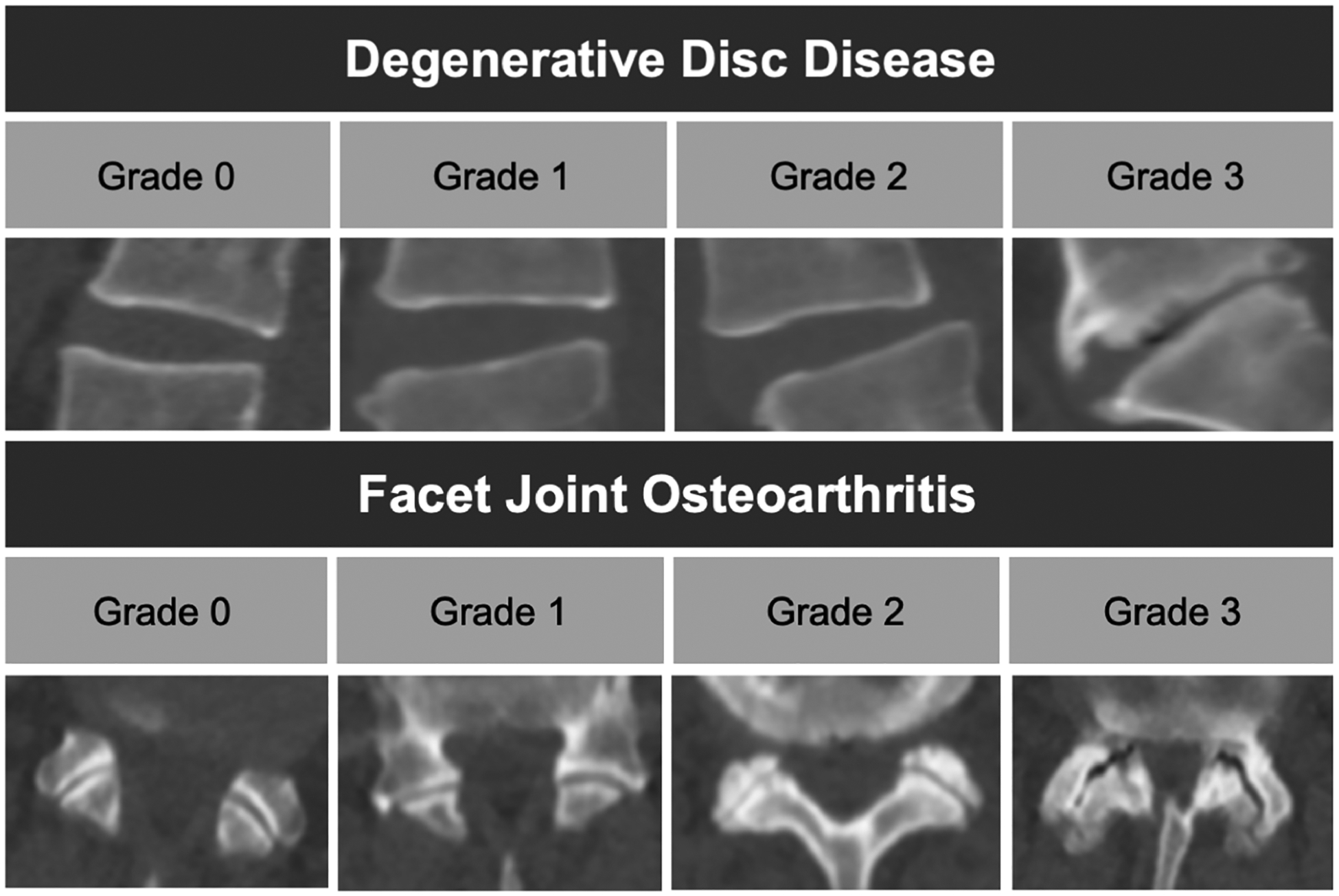
Representative CT images of grades 0–3 degenerative disc disease and facet joint osteoarthritis. Degenerative disc disease was assessed in the sagittal plane on a scale of 0 to 3, where 0 = normal, disc height greater than the cephalad disc (except for the L5–S1 disc); 1 = disc height equal to the cephalad disc if it is normal, with no to slight disc bulging; 2 = disc narrower than the cephalad disc if it is normal (generally associated with disc bulging anteriorly and/or posteriorly); and 3 = severe narrowing, with end plates almost in or in contact. Facet joint OA was quantified in the axial plane on a scale of 0 to 3, where 0 = normal; 1 = mild, with narrowing of the joint space (<2 mm) and small osteophytes and/or mild hypertrophy of the articular process; 2 = moderate, with narrowing of the joint space (<1 mm) and moderate osteophytes, moderate hypertrophy of the articular process, or mild subarticular bone erosions; and 3 = severe, with profound narrowing of the joint space and large osteophytes, severe hypertrophy of the articular process, severe subarticular bone erosions, or subchondral cysts.

Facet joint OA was quantified in the axial plane on a scale of 0 to 3, where 0 = normal; 1 = mild, with narrowing of the joint space (<2 mm) and small osteophytes and/or mild hypertrophy of the articular process; 2 = moderate, with narrowing of the joint space (<1 mm) and moderate osteophytes, moderate hypertrophy of the articular process, or mild subarticular bone erosions; and 3 = severe, with profound narrowing of the joint space and large osteophytes, severe hypertrophy of the articular process, severe subarticular bone erosions, or subchondral cysts (Figure 1).

Presence of spondylosis was defined in the sagittal and axial view as complete isthmic lysis, either uni- or bi-laterally. Presence of spondylolisthesis was defined in the sagittal view as loss of the posterior wall arciform line with a step >2 mm.

To dichotomize gradings at each level prior to statistical analysis, a subject was defined as having degenerative disc disease if they had a score of 2 or greater at any level, and as having facet joint OA if they had a score of 2 or greater at any level on either side. A subject was defined as having spondylosis if it was present at any level, and spondylolisthesis if it was present at any level. In addition to dichotomizing degenerative disc disease and facet joint OA, a mild/moderate/severe scale was also defined to assess for variable severity between subjects. In this definition, mild was defined as all 0s and 1s at all levels, severe was defined as 2s at all levels or 3 at any level, and moderate was defined as all else (e.g., a combo of 1s and 2s).

### 2.4. Analysis of Lumbosacral Monosodium Urate Crystal Deposition

Lumbosacral MSU load was quantified using DECT as previously outlined by our group [6] and as described further in the Supplemental Materials. Notably, we used both the manufacturer’s default post-processing settings and validated, maximally specific settings to better exclude potential artefacts when calculating spinal MSU-coded volumes [13]. Supplemental Figures S1 and S2 illustrate lumbosacral MSU and calcium deposition in a tophaceous gout subject and control subject as characterized by the default and more specific post-processing settings.

### 2.5. Statistical Analysis

Demographic characteristics of gout and control subjects were compared using Pearson’s chi-squared test and Fisher’s exact test for categorical variables and the Wilcoxon rank-sum test or Student’s t-test for continuous variables. Inter-rater reliability was assessed using Cohen’s kappa. Prevalence of degenerative disc disease, facet joint OA, spondylolysis, and spondylolisthesis, as well as severity of degenerative disc disease and facet joint OA on a mild/moderate/severe scale, were compared across gout and control subjects using Pearson’s chi-squared test and Fisher’s exact test. Logistic regression was used to adjust for potential confounders, including BMI, creatinine, hypertension, and exercise frequency. Significance was defined as a *p*-value < 0.05.

## 3. Results

### 3.1. Subjects

From 75 subjects enrolled in the original study, 71 (46 gout and 25 control subjects) were included in the final analysis. Four patients with gout were excluded: one was lost to follow-up prior to imaging, one had a DECT study *done* on a different scanner than other participants that was technically inadequate for the current analyses, and two were excluded for low SU measurements on repeat SU testing. For the analyses assessing MSU crystal deposition, an additional gout patient outlier with extremely elevated MSU was also excluded (Supplemental Figure S3). Mean age at study entry was 62 years, and 93% were men. Consistent with prior reports, patients with gout had greater incidence of chronic kidney disease and hypertension, higher BMI, and higher mean SU, creatinine, erythrocyte sedimentation rate, and C-reactive protein levels vs. control subjects. Gout subjects also exhibited lower exercise frequency compared with controls (Table 1).

### 3.2. Prevalence and Severity of Lumbosacral Osteoarthritis in Gout vs. Control Subjects

Inter-rater reliability was moderate (Table 2). There was high prevalence of facet joint OA (93%) and degenerative disc disease (92%) across all subjects. The prevalence of facet joint OA did not differ between gout subjects and controls (91% in the gout group vs. 96% in the control group, *p* = 0.65), nor did the prevalence of degenerative disc disease (89% in the gout group vs. 96% in the control group, *p* = 0.41). There was also no difference between groups in prevalence of spondylosis (6.5% in the gout group vs. 8.0% in the control group, *p* > 0.99) or spondylolisthesis (35% in the gout group vs. 40% in the control group, *p* = 0.66) (Table 3).

**Figure 2.**
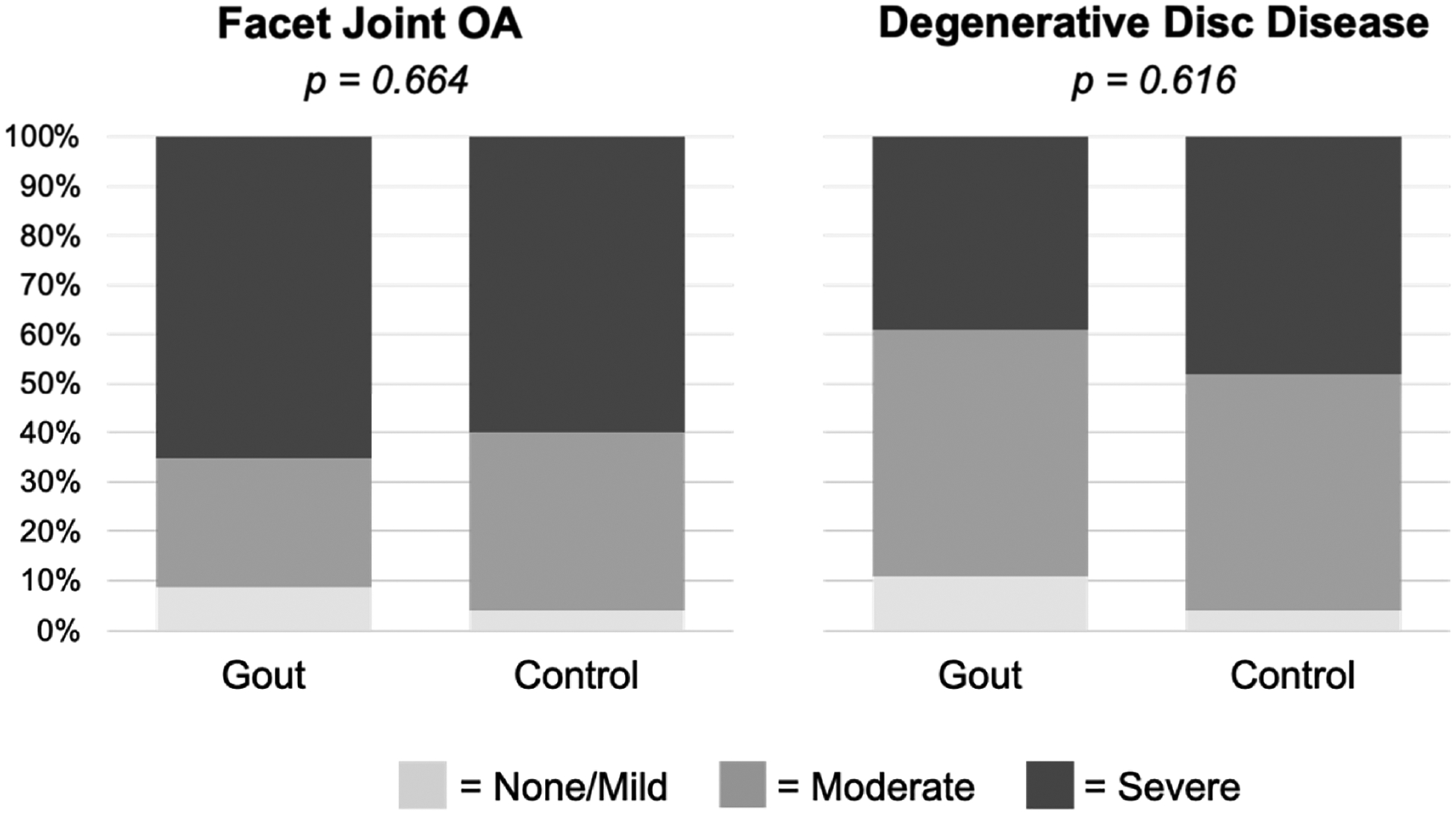
Lumbosacral osteoarthritis severity in gout subjects vs. controls. Bars show the percentage of gout or control subjects having mild vs. moderate vs. severe facet joint osteoarthritis (OA) and degenerative disc disease. Mild was defined as no or mild OA at all levels, severe was defined as severe OA at any level or moderate OA at all levels, and moderate was defined as all else. *p*-values were generated using Pearson’s chi-squared test.

When facet joint OA and degenerative disc disease were assessed by severity, there was no difference between gout patients and controls in the proportion of mild vs. moderate vs. severe facet joint OA (4% mild in the gout group vs. 9% in the control group, 36% moderate in the gout group vs. 26% in the control group, 60% severe in the gout group vs. 65% in the control group, *p* = 0.66). There was also no difference in mild vs. moderate vs. severe degenerative disc disease (4% mild in the gout group vs. 11% in the control group, 48% moderate in the gout group vs. 50% in the control group, 48% severe in the gout group vs. 39% in the control group, *p* = 0.41) (Figure 2).

### 3.3. Hyperuricemia vs. Normouricemia and Tophaceous vs. Non-Tophaceous Gout

Additional analyses assessed LS OA prevalence in subjects with hyperuricemia (serum urate > 6.8) vs. normouricemia, and tophaceous vs. non-tophaceous gout subjects. The prevalence of facet joint OA did not differ between hyperuricemic and normouricemic subjects (92% in the hyperuricemic group vs. 94% in the normouricemic group, *p* > 0.99), nor did the prevalence of degenerative disc disease (89% in the hyperuricemic group vs. 94% in the normouricemic group, *p* = 0.68). There was also no difference between groups in prevalence of spondylosis (8% in the hyperuricemic group vs. 6% in the normouricemic group, *p* > 0.99) or spondylolisthesis (39% in the hyperuricemic group vs. 33% in the normouricemic group, *p* = 0.59) (Supplemental Table S1).

Comparing tophaceous and non-tophaceous gout subjects, we did see a higher prevalence of spondylolisthesis in tophaceous gout subjects (52% in the tophaceous group vs. 20% in the non-tophaceous group, *p* = 0.02), as well as a trend towards higher prevalence of degenerative disc disease (100% in the tophaceous group vs. 80% in the non-tophaceous group, *p* = 0.054). However, there was no difference between groups in prevalence of facet joint OA (95% in the tophaceous group vs. 88% in the non-tophaceous group, *p* = 0.61), or spondylolysis (10% in the tophaceous group vs. 4% in the non-tophaceous group, *p* = 0.59) (Supplemental Table S2).

### 3.4. Lumbosacral Monosodium Urate Crystal Deposition and Osteoarthritis

**Figure 3.**
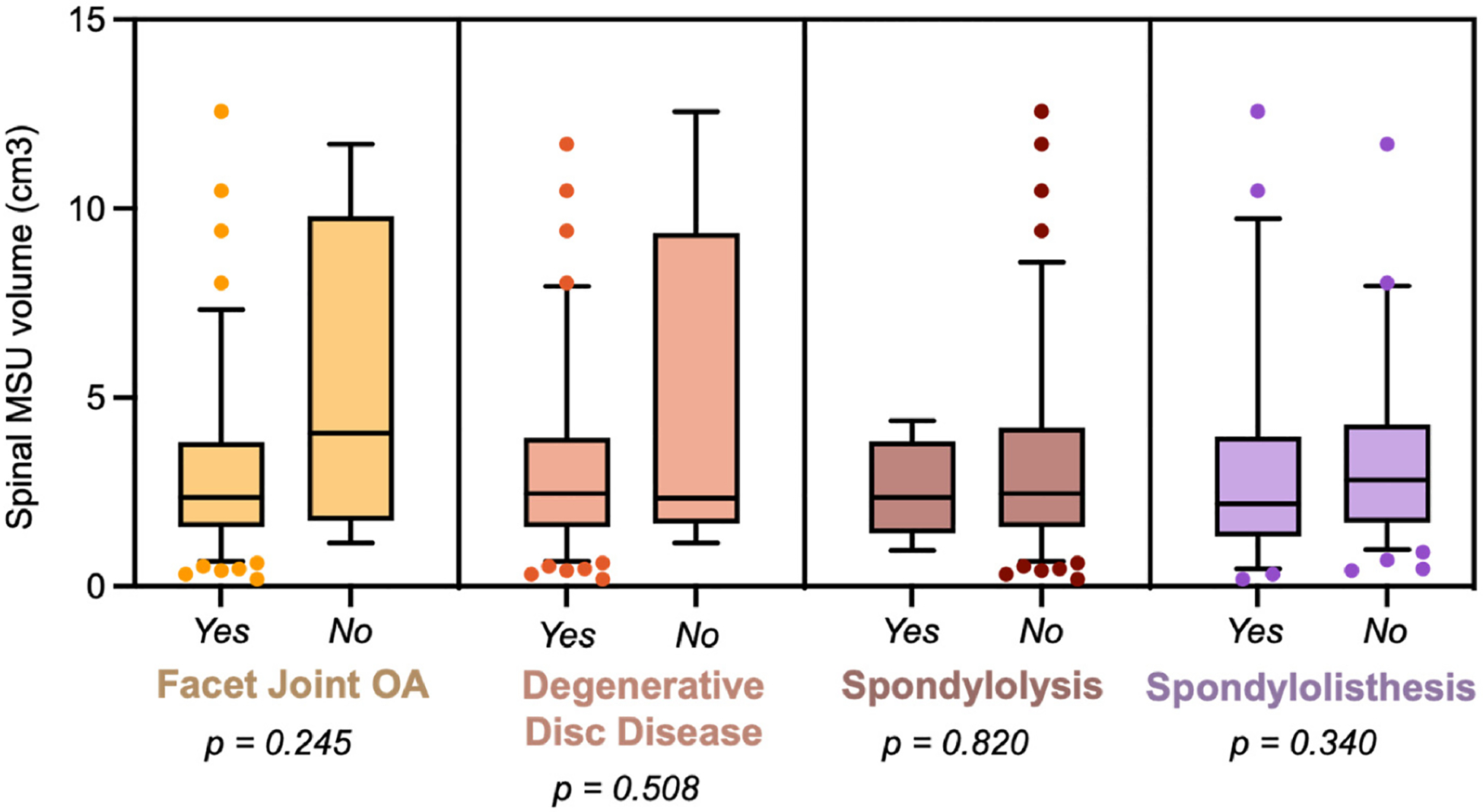
Monosodium urate crystal deposition in subjects with vs. without lumbosacral osteoarthritis. All plots show median, interquartile range, and minimum and maximum values excluding outliers, which are defined as >1.5 times the interquartile range and shown as individual data points. Osteoarthritis (OA) parameters were defined as yes for degenerative disc disease if 2 or greater at any level, for facet joint OA if 2 or greater at any level on either side, for spondylosis if present at any level, and for spondylolisthesis if present at any level. Lumbosacral spinal monosodium urate (MSU) volume was measured in cubic centimetres using dual-energy computed tomography and calculated using default post-processing settings, with subsequent exclusion of one gout outlier with extremely high MSU deposition in the analyses.

When MSU deposition was quantified using manufacturer’s default post-processing settings, there was no difference in volume of LS MSU crystal deposition in subjects with vs. without facet joint OA (median 2.35 cm^3^, interquartile range [IQR] 1.56–3.82 in subjects with facet joint OA vs. median 4.05 cm^3^, IQR 1.74–9.80 in subjects without). There was also no difference in MSU deposition in patients with vs. without degenerative disc disease (median 2.45 cm^3^, IQR 1.56–3.94 in subjects with degenerative disc disease vs. median 2.33 cm^3^, IQR 1.67–9.36 in subjects without), spondylolysis (median 2.35 cm^3^, IQR 1.40–3.85 in subjects with spondylolysis vs. median 2.45 cm^3^, IQR 1.56–4.21 in subjects without), or spondylolisthesis (median 2.19 cm^3^, IQR 1.56–3.82 in subjects with spondylolisthesis vs. median 2.82 cm^3^, 1.68–4.28 in subjects without) (Figure 3). When MSU was quantified using a validated, maximally specific algorithm to exclude potential artefacts, there remained no difference in LS MSU deposition in subjects with vs. without facet joint OA, degenerative disc disease, spondylolysis, or spondylolisthesis (Supplemental Table S3).

## 4. Discussion

In this study, we did not find a higher prevalence of LS OA in subjects with gout vs. non-gout controls. Subjects with hyperuricemia also did not have a higher prevalence of LS OA compared to normouricemics. Additionally, subjects with LS OA did not have higher levels of spinal MSU crystal deposition vs. subjects without LS OA. Tophaceous gout subjects did have higher prevalence of spondylolisthesis and a trend towards more degenerative disc disease, though they did not have higher prevalence of facet joint OA or spondylolysis.

While prior work suggests that the spine does have MSU deposition [6,19], it does not appear to be a common site of gout flares. Thus, our findings suggest that at sites not commonly affected by gouty attacks, such as the LS spine, we may not see a relationship between gout and OA. The proposed pathophysiologic factors connecting MSU deposition and OA are complex and include activation of the NLRP3 inflammasome, nitric oxide production, and chondrocyte death via a non-apoptotic process [20,21]. However, other research suggests that some degree of urate may actually be protective against OA and promote collagen secretion [22]. These findings together suggest that MSU deposition in the spine without the additional inflammatory cascade of a gout flare may not lead to significant chondrocyte damage or death and subsequent OA potentiation.

We did see higher prevalence of spondylolisthesis and a trend towards higher prevalence of degenerative disc disease in tophaceous gout subjects as compared with non-tophaceous gout subjects. These analyses were exploratory given our limited sample size (25 non-tophaceous and 21 tophaceous subjects), and the fact that we need to account for testing multiple hypotheses when interpreting our *p*-values. As described previously by our group [6], the tophaceous gout participants in this study did not have differing serum urate nor spinal MSU deposition vs. non-tophaceous participants, and they actually had lower BMI than non-tophaceous participants, indicating that if there is a relationship between tophaceous gout and LS OA it does not seem to be mediated by MSU deposition, nor confounded by a higher BMI in these patients. Nonetheless, these findings did suggest that there may be a relationship between tophaceous gout specifically and LS OA that could be explored through additional research.

In our study, the prevalence of LS OA was substantial in both the gout and control groups. While we did attempt to overcome this limitation by stratifying by severity, there remained a high degree of severe LS OA in both groups. Given the average age of 62 years in both gout and control groups, the high prevalence of radiological LS OA was not surprising, but it is possible that our results were subject to a ‘ceiling effect’, whereby the outcome of LS OA was too prevalent in both groups to detect a meaningful difference. To overcome this limitation, additional research could assess younger gout subjects to see if they experience earlier onset of LS OA versus non-gout controls.

In our group’s original study assessing MSU deposition in the spines of gout patients, we did find a trend (not statistically significant) towards more lower back pain in gout patients vs. non-gout controls [6]. Based on the results of the present study, an increase in back pain in gout patients may not be explained by more radiographically severe LS OA but may relate to higher levels of obesity, lower exercise frequency, or other factors.

Limitations of our study include the relatively small sample size, and the fact that our study enrolled mainly White and Black men over the age of 60, potentially limiting its generalizability to other genders, ages, races and ethnic groups. This study was also an ancillary analysis to a prior study from our group, so the enrollment and data collection were not designed specifically to assess for LS OA in patients with gout. We did not assess the role of co-existing calcium crystal deposition, which may also contribute to OA. We also were not able to assess whether there were localized sites of disproportionate spinal degenerative disease at sites of highest MSU concentration, as we had data on whole LS MSU deposition volume but not on MSU load per disc level or joint.

Our study also had several strengths. To our knowledge, this is the first study to directly explore the relationship between gout and LS OA, adding to the body of literature on gout and OA at other sites such as the knee, ankle, and hand [10,11,23]. Our study included both tophaceous and non-tophaceous gout patients, allowing us to assess OA and MSU deposition across the two subsets. Additionally, our use of DECT allowed us to not only assess for differences in LS OA between gout and control participants but also to assess the relationship of LS OA to MSU crystal deposition directly. We recognize that spinal MSU volumes obtained using DECT with default post-processing settings are likely to contain artefacts and should be interpreted with caution, but we also utilized more stringent, previously validated post-processing settings to exclude potential artefacts [13], and confirmed that our findings were consistent across both post-processing settings.

In summary, in this study LS OA was not more prevalent or more severe in people with gout compared with controls, and spinal MSU crystal deposition did not differ between subjects with and without LS OA. These findings suggest that gout and MSU deposition may not play a role in the development of radiological OA in joints not commonly affected by gouty attacks. Additional research on younger patients without a substantial prevalence of baseline LS OA, as well as on a larger sample of tophaceous gout patients specifically, may be able to better determine whether patients with gout are at risk of premature LS OA.

## Supplementary Material

CovelloEtAl.Supp

The following supporting information can be downloaded at: https://www.mdpi.com/article/10.3390/gucdd4020010/s1, Supplemental Methods, Supplemental Figure S1: Representative lumbosacral DECT images of tophaceous gout subject using default manufacturer post-processing and specific post-processing settings, Supplemental Figure S2: Representative lumbosacral DECT images of control subject using default manufacturer post-processing and specific post-processing settings, Supplemental Figure S3: Flowchart of participant selection, Supplemental Table S1: Lumbosacral osteoarthritis prevalence in hyperuricemic versus normouricemic subjects, Supplemental Table S2: Lumbosacral osteoarthritis prevalence in tophaceous versus non-tophaceous gout subjects, Supplemental Table S3: MSU deposition in subjects with versus without lumbosacral osteoarthritis (calculated using maximally specific post-processing algorithm).

## Figures and Tables

**Table 1.** Baseline sample size and cohort characteristics.

Variable	Gout n = 46	Control n = 25	*p*-Value
Age (years)	63 ± 5	62 ± 3	0.57
Male sex (n, %)	43 (93%)	23 (92%)	1.00
Race and ethnicity (n, %)			0.36
White	24 (52%)	18 (72%)	
Black	14 (30%)	6 (24%)	
Hispanic or Latino/a/x	6 (13%)	1 (4%)	
Asian or Pacific Islander	2 (4%)	0 (0%)	
BMI (kg/m^2^)	30.75 [27.55–34.68]	26.50 [24.80–30.00]	<0.01
Medical conditions (n, %)			
Hypertension	33 (72%)	6 (24%)	<0.001
Chronic kidney disease	12 (26%)	0 (0%)	<0.01
Diabetes mellitus type 2	9 (20%)	3 (12%)	0.52
Hyperlipidemia	29 (63%)	14 (56%)	0.56
History of myocardial infarction	4 (9%)	1 (4%)	0.65
History of cancer	6 (13%)	3 (12%)	1.00
Serum levels			
Urate (mg/dL)	8.70 [7.18–9.50]	5.20 [4.50–6.10]	<0.001
Creatinine (mg/dL)	1.23 [1.09–1.51]	0.92 [0.85–1.11]	<0.001
ESR (mm/h)	24.0 [13.25–39.0]	9.0 [4.0–16.0]	<0.01
hsCRP (mg/L)	3.60 [1.60–7.28]	1.20 [0.60–2.30]	<0.01
Exercise frequency			<0.05
0 times per week	27 (59%)	7 (28%)	
1–2 times per week	8 (17%)	6 (24%)	
3+ times per week	11 (24%)	12 (48%)	

Abbreviations: BMI = body mass index, ESR = erythrocyte sedimentation rate, hsCRP = C-reactive protein. Note: Values are mean ± standard deviation, n (%), or median [interquartile range]. *p*-values were generated using Pearson’s Chi-squared test, Fisher’s exact test, Wilcoxon rank-sum test or Wilcoxon rank-sum exact test. Race and ethnicity are defined based on participant self-report, using participants’ primary response.

**Table 2.** Two-rater interobserver reliabilities.

	Observed Agreement (%)	Expected Agreement (%)	Cohen’s Kappa	Standard Error	*p*-Value
Facet joint osteoarthritis	93.0%	87.9%	0.42	0.10	<0.001
Degenerative disc disease	78.6%	65.9%	0.37	0.11	<0.001
Spondylolysis	91.5%	84.3%	0.46	0.11	<0.001
Spondylolisthesis	77.5%	48.3%	0.56	0.11	<0.001

**Table 3.** Lumbosacral osteoarthritis prevalence in gout subjects vs. controls.

Features	Gout n = 46	Control n = 25	*p*-Value
Facet joint osteoarthritis	42 (91%)	24 (96%)	0.650
Degenerative disc disease	41 (89%)	24 (96%)	0.414
Spondylolysis	3 (7%)	2 (8%)	>0.999
Spondylolisthesis	16 (35%)	10 (40%)	0.663

Values are n (%). *p*-values were generated using Pearson’s Chi-squared test and Fisher’s exact test.

## Data Availability

The imaging dataset used in this study is available upon request to the corresponding author. For privacy reasons, these images are not publicly available.

## Abbreviations

The following abbreviations are used in this manuscript:

OA: Osteoarthritis
LS: Lumbosacral
DECT: Dual-energy computed tomography
MSU: Monosodium urate
IQR: Inter-quartile range
ESR: Erythrocyte sedimentation rate
hsCRP: C-reactive protein
