# Supplementary material for: Prevalence and Severity of Spinal Osteoarthritis in Gout Patients Versus Non-Gout Controls
Source: Gout Urate Cryst Depos Dis. Author manuscript; Available in PMC 2026 Aug 18. (PMC13480402; doi:10.3390/gucdd4020010)
Supplement: Supplementary file 1 [file NIHMS2203554-supplement-CovelloEtAl_Supp.pdf]

Supplemental Materials

Supplemental Methods

Analysis of MSU Deposition

Lumbosacral MSU load was quantified from DECT scans post-processed using the gout application profile in a proprietary software package (syngo.via VB30A; Siemens Healthineers) by two musculoskeletal radiologists. First, one radiologist calculated spinal MSU volume with the manufacturer's gout preset (i.e., dual-energy ratio [DER], 1.25; minimum HU, 150; resolution, 5). With the aim to identify only voxels definitively representing MSU while excluding other chemical element/compounds mimicking MSU attenuation (e.g. low-concentration calcium deposition, aged fibrocartilage, or hyaline cartilage), a second radiologist (FB) repeated the analysis using thresholds tuned to pure MSU (i.e., voxels included within the 95% confidence interval of the DER characteristic for synthetic MSU [0.99, 1.05] with this specific DECT scanner and acquisition protocol) as previously described. In brief, the DER or dual-energy index, which reflects the electron density (Rho) and effective atomic number (Zeff) values of MSU or any other specific material, was adjusted to optimized dual thresholds (i.e., upper and lower bounds), which were then measured for a sample of cases to confirm the accuracy in post-processing settings. The vast majority of typical DECT artifacts, including vascular (e.g. abdominal aorta) signal disappeared entirely with these settings. These optimized settings have been published for the same hardware and protocol [1], and both readers were blinded to all clinical data. While highly specific, these optimized settings may also exclude some true gout signal.

1. Døssing A, Müller FC, Becce F, et al. Dual-energy computed tomography for detection and characterization of monosodium urate, calcium pyrophosphate, and hydroxyapatite: a phantom study on diagnostic performance. *Invest Radiol* 2021;56(7):417–24.

Supplemental Figure S1: Representative lumbosacral DECT images of tophaceous gout subject using default manufacturer post-processing and specific post-processing settings. Green highlights represent MSU-consistent signal, and purple represents calcification. The specific post-processing settings exclude potential artifact and mischaracterization of calcium as MSU. A small amount of MSU-consistent signal persists in the specific post-processing images, indicating the presence of MSU deposition.

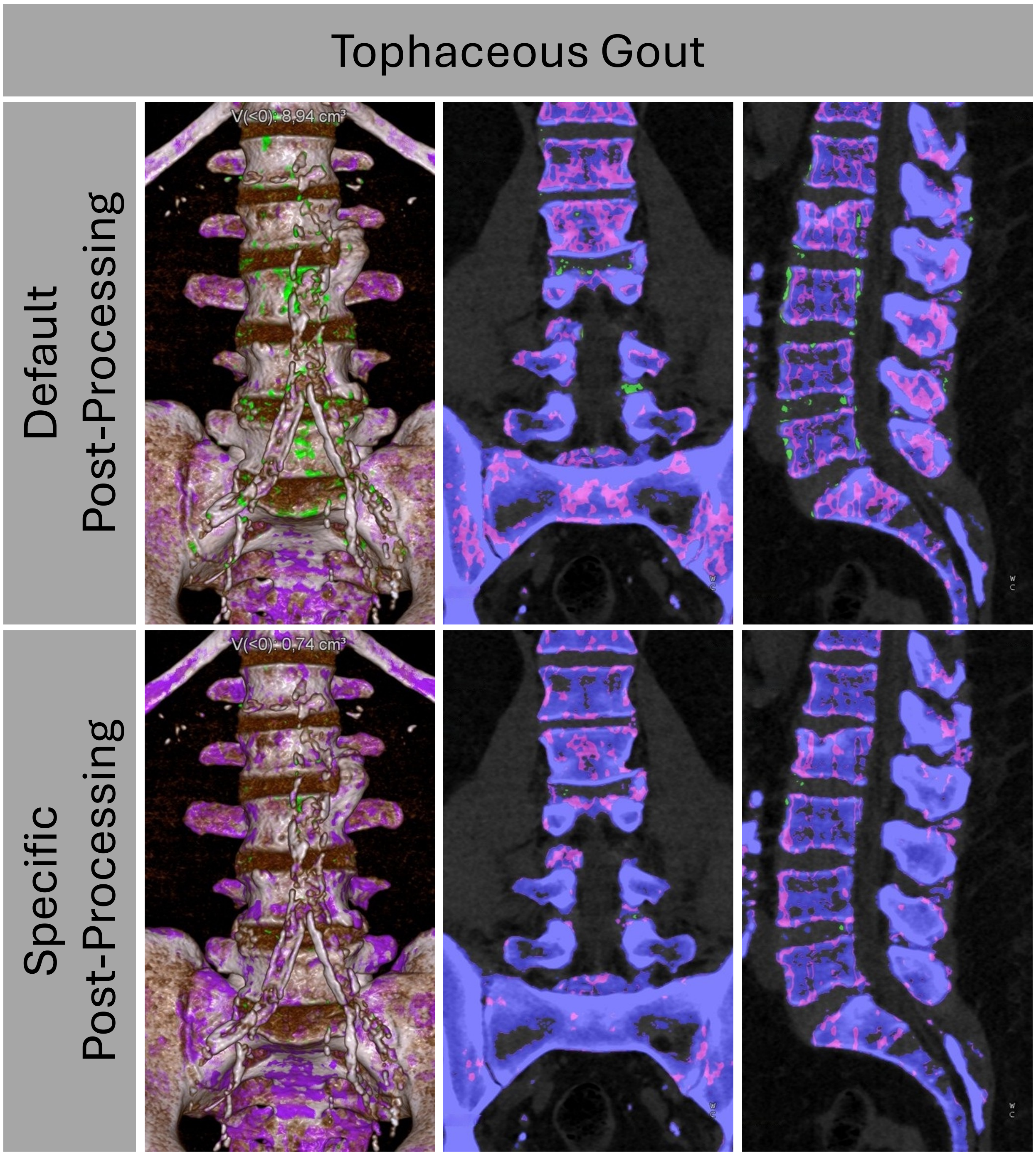

Supplemental Figure S2: Representative lumbosacral DECT images of control subject using default manufacturer post-processing and specific post-processing settings. Green highlights represent MSU-consistent signal, and purple represents calcification. The specific post-processing settings exclude potential artifact and mischaracterization of calcium as MSU. No MSU-consistent signal is observed after specific post-processing, indicating that signal using default processing was likely artifact.

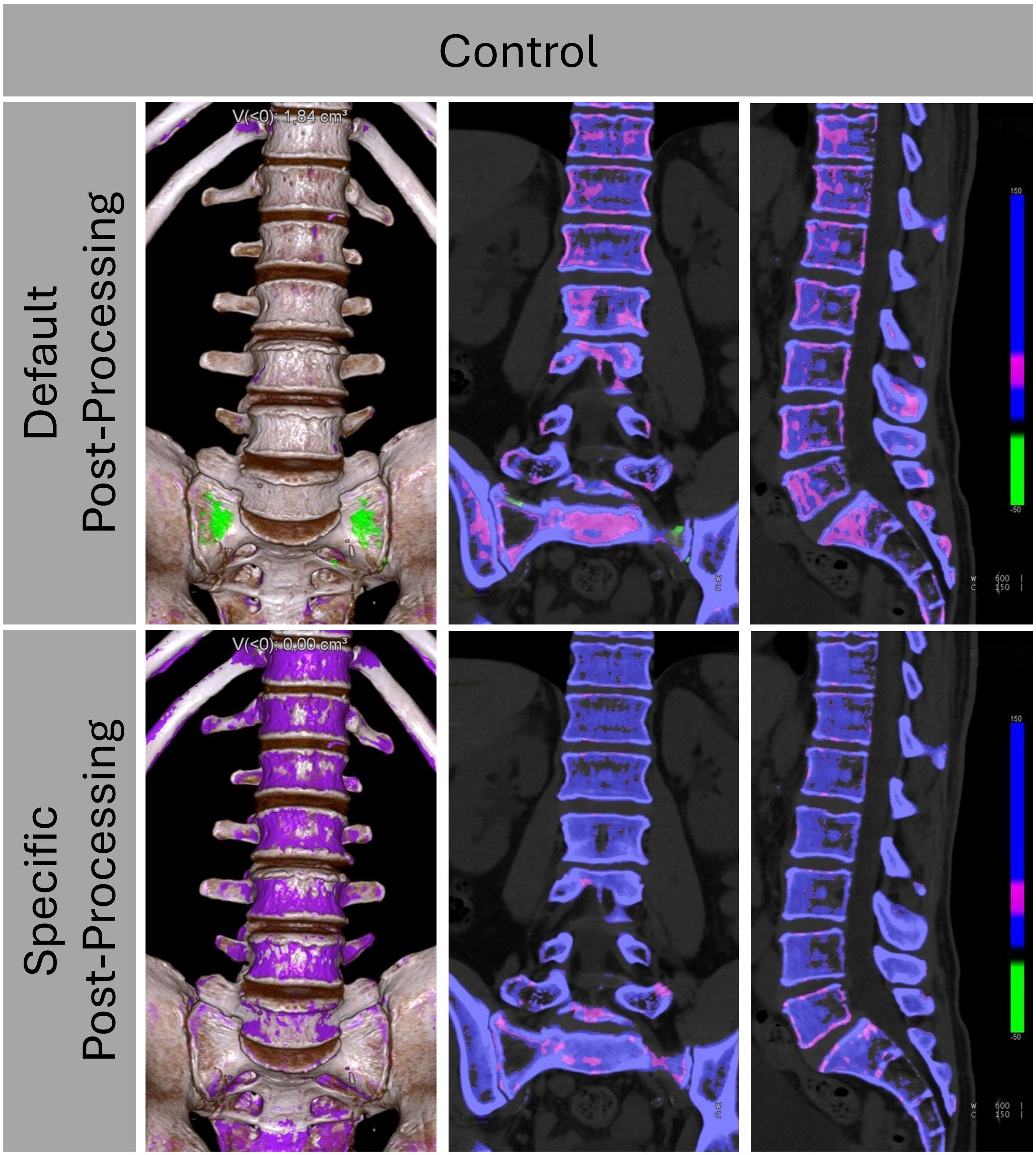

Supplemental Figure S3: Flowchart of participant selection

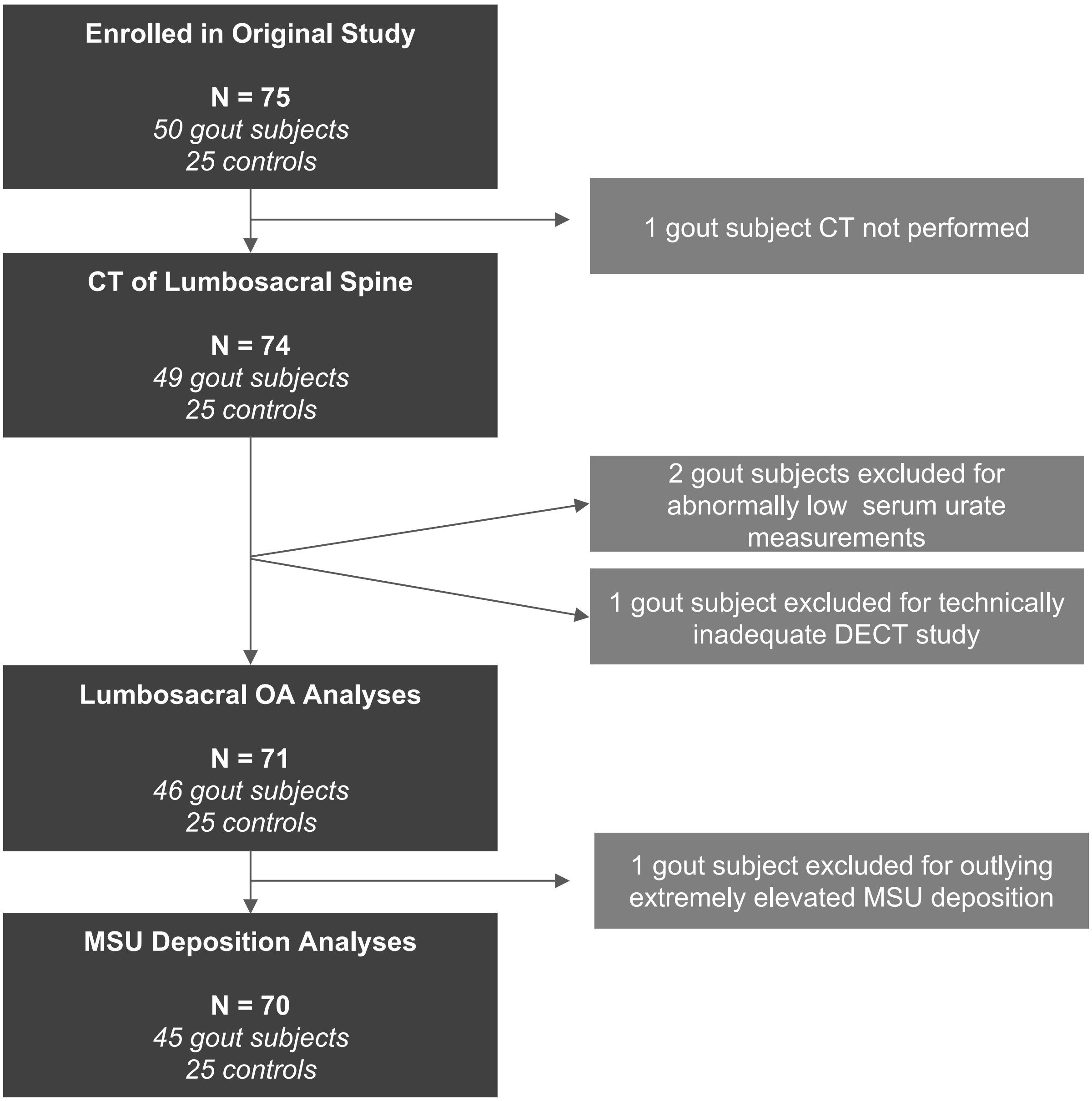

Supplemental Table S1: Lumbosacral osteoarthritis prevalence in hyperuricemic versus normouricemic subjects

Features	Normouricemic n=33	Hyperuricemic n=38	P-value
Facet joint OA	31 (94%)	35 (92%)	>0.999
Degenerative Disc Disease	31 (94%)	34 (89%)	0.679
Spondylolysis	2 (6.1%)	3 (7.9%)	>0.999
Spondylolisthesis	11 (33%)	15 (39%)	0.592

Values are n (%). P-values were generated using Pearson's Chi-squared test and Fisher's exact test.

Supplemental Table S2: Lumbosacral osteoarthritis prevalence in tophaceous versus non-tophaceous gout subjects

Features	Non- Tophaceous Gout n=25	Tophaceous Gout n=21	P-value
Facet joint OA	22 (88%)	20 (95%)	0.614
Degenerative Disc Disease	20 (80%)	21 (100%)	0.054
Spondylolysis	1 (4.0%)	2 (9.5%)	0.585
Spondylolisthesis	5 (20%)	11 (52%)	0.022

Values are n (%). P-values were generated using Pearson's Chi-squared test and Fisher's exact test.

Supplemental Table S3: MSU deposition in subjects with versus without lumbosacral osteoarthritis (calculated using maximally specific post-processing algorithm)

Features	Yes	No	P-value
Facet joint OA	0.03 cm^3 [0.01-0.08] (n=65)	0.08 cm^3 [0.02-0.14] (n=5)	0.390
Degenerative Disc Disease	0.03 cm^3 [0.01-0.08] (n=65)	0.01 cm^3 [0.01-0.08] (n=5)	0.740
Spondylolysis	0.05 cm^3 [0.00-0.10] (n=5)	0.03 cm^3 [0.00-3.81] (n=65)	0.688
Spondylolisthesis	0.03 cm^3 [0.01-0.08] (n=26)	0.04 cm^3 [0.01-0.08] (n=44)	0.549

Values are median [IQR], and p-values were generated using Wilcoxon rank-sum test and Wilcoxon rank-sum exact test.
